# Supplementary material for: A Likelihood Approach for Real-Time Calibration of Stochastic Compartmental Epidemic Models
Source: PLoS Comput Biol. 2017 Jan 17;13(1):e1005257. doi: 10.1371/journal.pcbi.1005257 (PMC5240920; doi:10.1371/journal.pcbi.1005257)
Supplement: S1 File — (TAR.GZ) [file pcbi.1005257.s014.tar.gz › HSPH_Online-SI-Revision/EnKF07/n1-severe/n1-severe_EnKF2_table-4.pdf]

1	newly 3 weeks spec	33.	12.97	{0., 23.}	97.1212	1.45124	newly 3 weeks cumul	81.	129.95	{8., 418.}	145.469	0.494226	newly 5 weeks spec	34.	6.17	{0., 11.}	98.4412	1.95073	newly 5 weeks cumul	158.	145.04	{9., 523.}	109.266	0.649361	newly 58 weeks cumul	531.	158.09	{9., 523.}	86.0207	1.02018
2	newly 3 weeks spec	55.	22.53	{0., 51.}	98.7091	1.356	newly 3 weeks cumul	130.	155.57	{11., 553.}	120.038	0.5506	newly 5 weeks spec	53.	8.57	{0., 27.}	92.1698	1.90169	newly 5 weeks cumul	247.	177.83	{11., 758.}	98.1822	0.706764	newly 60 weeks cumul	592.	194.25	{11., 871.}	83.3193	0.963981
3	newly 3 weeks spec	32.	16.83	{0., 49.}	104.594	1.119	newly 3 weeks cumul	74.	119.84	{8., 374.}	148.838	0.502579	newly 5 weeks spec	43.	4.93	{0., 15.}	91.4186	1.80474	newly 5 weeks cumul	164.	133.4	{8., 417.}	108.012	0.705877	newly 59 weeks cumul	553.	141.47	{8., 421.}	85.5063	1.07646
4	newly 3 weeks spec	58.	30.07	{2., 81.}	78.1897	0.731908	newly 3 weeks cumul	144.	231.64	{49., 484.}	102.306	0.296213	newly 5 weeks spec	59.	10.01	{0., 25.}	86.8305	1.46386	newly 5 weeks cumul	286.	258.1	{62., 589.}	74.4266	0.393861	newly 62 weeks cumul	620.	283.12	{63., 623.}	68.271	0.580383
5	newly 3 weeks spec	56.	11.38	{0., 24.}	85.7857	1.3178	newly 3 weeks cumul	140.	140.18	{17., 361.}	72.3143	0.38205	newly 5 weeks spec	40.	2.99	{0., 8.}	93.525	1.91689	newly 5 weeks cumul	241.	148.98	{17., 417.}	69.3859	0.519102	newly 61 weeks cumul	534.	152.58	{17., 417.}	74.4607	0.782674
6	newly 3 weeks spec	31.	7.79	{0., 12.}	97.8387	1.62822	newly 3 weeks cumul	71.	82.54	{6., 81.}	114.676	0.525234	newly 5 weeks spec	40.	3.57	{0., 8.}	95.775	2.17095	newly 5 weeks cumul	159.	90.77	{6., 113.}	98.5975	0.791891	newly 58 weeks cumul	620.	95.92	{6., 114.}	90.9903	1.29963
7	newly 3 weeks spec	37.	13.09	{0., 21.}	97.9189	1.57832	newly 3 weeks cumul	101.	111.55	{9., 156.}	122.089	0.568433	newly 5 weeks spec	30.	3.94	{0., 10.}	94.4667	1.87749	newly 5 weeks cumul	170.	122.24	{9., 186.}	106.082	0.719489	newly 58 weeks cumul	392.	127.82	{9., 194.}	92.199	1.00115
8	newly 3 weeks spec	83.	29.24	{2., 90.}	80.6747	0.918056	newly 3 weeks cumul	181.	218.	{42., 591.}	85.4696	0.333408	newly 5 weeks spec	97.	10.38	{0., 22.}	90.6804	1.81812	newly 5 weeks cumul	384.	245.39	{47., 626.}	73.2891	0.500761	newly 62 weeks cumul	588.	274.93	{51., 710.}	73.4881	0.614463
9	newly 3 weeks spec	38.	7.85	{0., 11.}	95.8684	1.7651	newly 3 weeks cumul	79.	82.25	{6., 103.}	111.101	0.568164	newly 5 weeks spec	31.	2.58	{0., 4.}	97.9355	2.1133	newly 5 weeks cumul	150.	88.28	{7., 117.}	97.8267	0.773962	newly 58 weeks cumul	454.	92.05	{7., 121.}	90.9273	1.17873
10	newly 3 weeks spec	67.	5.12	{0., 11.}	93.403	1.90915	newly 3 weeks cumul	156.	84.18	{7., 94.}	92.9872	0.762147	newly 5 weeks spec	47.	2.32	{0., 5.}	96.	2.23344	newly 5 weeks cumul	273.	89.78	{7., 105.}	89.9048	0.947092	newly 58 weeks cumul	540.	93.08	{7., 114.}	89.3852	1.20074
11	newly 3 weeks spec	25.	6.57	{0., 11.}	96.92	1.66165	newly 3 weeks cumul	76.	108.9	{10., 424.}	140.947	0.556241	newly 5 weeks spec	31.	3.1	{0., 5.}	94.5806	2.00566	newly 5 weeks cumul	146.	116.82	{11., 425.}	104.589	0.703922	newly 55 weeks cumul	505.	126.65	{11., 476.}	78.9683	1.03835
12	newly 3 weeks spec	95.	39.2	{0., 87.}	73.7895	0.85542	newly 3 weeks cumul	232.	330.05	{78., 874.}	84.5129	0.295582	newly 5 weeks spec	59.	11.	{0., 23.}	85.2881	1.38302	newly 5 weeks cumul	391.	360.1	{93., 983.}	66.0921	0.354968	newly 63 weeks cumul	569.	378.18	{102., 983.}	64.6819	0.439168
13	newly 3 weeks spec	46.	7.81	{0., 20.}	89.3696	1.66781	newly 3 weeks cumul	111.	78.58	{8., 88.}	93.4595	0.607142	newly 5 weeks spec	49.	2.79	{0., 7.}	94.7551	2.19811	newly 5 weeks cumul	221.	85.43	{9., 102.}	90.9095	0.845982	newly 58 weeks cumul	585.	89.92	{9., 113.}	89.9419	1.20898
14	newly 3 weeks spec	59.	36.61	{0., 71.}	67.7458	0.722012	newly 3 weeks cumul	122.	308.18	{102., 712.}	162.082	0.352833	newly 5 weeks spec	75.	10.25	{0., 28.}	86.3333	1.44381	newly 5 weeks cumul	272.	337.32	{107., 810.}	64.0662	0.247467	newly 63 weeks cumul	614.	352.39	{121., 850.}	60.0147	0.424895
15	newly 3 weeks spec	43.	5.92	{0., 11.}	90.6512	1.56896	newly 3 weeks cumul	132.	89.39	{5., 81.}	109.144	0.788973	newly 5 weeks spec	36.	2.91	{0., 5.}	95.4722	1.95009	newly 5 weeks cumul	229.	95.81	{5., 122.}	99.1485	0.961252	newly 57 weeks cumul	373.	107.48	{5., 122.}	94.1609	1.10193
16	newly 3 weeks spec	37.	35.83	{0., 79.}	80.2973	0.653057	newly 3 weeks cumul	113.	293.8	{90., 694.}	169.487	0.366114	newly 5 weeks spec	55.	11.05	{0., 32.}	80.8909	1.36281	newly 5 weeks cumul	226.	324.62	{109., 776.}	73.885	0.245601	newly 63 weeks cumul	577.	348.28	{132., 822.}	58.3986	0.402647
17	newly 3 weeks spec	80.	35.84	{0., 67.}	75.025	0.893596	newly 3 weeks cumul	176.	296.48	{87., 757.}	96.6364	0.287123	newly 5 weeks spec	63.	11.45	{0., 33.}	84.3968	1.46773	newly 5 weeks cumul	335.	326.87	{104., 757.}	60.994	0.310839	newly 63 weeks cumul	583.	349.3	{112., 826.}	62.6209	0.452086
18	newly 3 weeks spec	27.	43.64	{2., 76.}	121.185	0.518237	newly 3 weeks cumul	67.	315.32	{80., 677.}	376.597	0.580799	newly 5 weeks spec	23.	13.3	{0., 32.}	86.4348	1.00601	newly 5 weeks cumul	126.	351.44	{102., 789.}	189.111	0.390302	newly 63 weeks cumul	593.	371.34	{114., 858.}	62.2091	0.4321
19	newly 3 weeks spec	46.	15.18	{0., 32.}	97.	1.45371	newly 3 weeks cumul	143.	139.39	{5., 279.}	129.699	0.810289	newly 5 weeks spec	45.	4.98	{0., 14.}	93.3333	2.03788	newly 5 weeks cumul	247.	152.28	{6., 488.}	105.158	0.936921	newly 56 weeks cumul	533.	159.03	{6., 537.}	89.6417	1.17021
20	newly 3 weeks spec	63.	11.52	{0., 23.}	93.1111	1.58803	newly 3 weeks cumul	168.	73.7	{9., 121.}	90.7024	0.823525	newly 5 weeks spec	55.	4.54	{0., 9.}	94.0727	2.10073	newly 5 weeks cumul	291.	84.39	{9., 158.}	88.6014	0.989239	newly 56 weeks cumul	494.	99.71	{9., 206.}	87.4352	1.15658
21	newly 3 weeks spec	38.	36.71	{0., 74.}	79.3421	0.605568	newly 3 weeks cumul	97.	275.12	{89., 498.}	192.268	0.388751	newly 5 weeks spec	50.	11.87	{0., 35.}	81.26	1.34034	newly 5 weeks cumul	199.	307.41	{106., 498.}	82.8693	0.25809	newly 63 weeks cumul	538.	333.92	{119., 781.}	60.1041	0.414213
22	newly 3 weeks spec	77.	12.58	{0., 20.}	92.1039	1.47102	newly 3 weeks cumul	210.	90.56	{5., 133.}	96.1905	0.88999	newly 5 weeks spec	61.	7.5	{0., 13.}	95.1148	1.88583	newly 5 weeks cumul	359.	107.79	{6., 156.}	92.0195	1.02548	newly 57 weeks cumul	537.	137.15	{6., 222.}	89.7933	1.10551
23	newly 3 weeks spec	30.	16.26	{0., 46.}	102.2	1.06954	newly 3 weeks cumul	92.	107.44	{7., 246.}	120.565	0.573672	newly 5 weeks spec	37.	5.68	{0., 7.}	92.5946	1.82961	newly 5 weeks cumul	174.	122.47	{7., 343.}	101.707	0.742295	newly 59 weeks cumul	461.	133.72	{7., 413.}	86.7592	1.04094
24	newly 3 weeks spec	38.	21.63	{0., 46.}	112.289	1.37898	newly 3 weeks cumul	121.	115.08	{6., 335.}	123.355	0.816316	newly 5 weeks spec	32.	5.53	{0., 10.}	92.0938	1.81353	newly 5 weeks cumul	201.	131.73	{6., 517.}	106.652	0.941221	newly 56 weeks cumul	407.	138.59	{6., 582.}	91.0885	1.15597
25	newly 3 weeks spec	55.	5.57	{0., 10.}	93.2545	1.66547	newly 3 weeks cumul	180.	48.93	{12., 94.}	86.7944	0.898602	newly 5 weeks spec	62.	2.04	{0., 6.}	96.7097	2.1867	newly 5 weeks cumul	314.	54.01	{15., 114.}	87.6146	1.07537	newly 54 weeks cumul	547.	58.05	{15., 123.}	89.8702	1.27345
26	newly 3 weeks spec	41.	12.13	{0., 28.}	82.5122	1.15007	newly 3 weeks cumul	123.	139.1	{17., 303.}	74.0325	0.354593	newly 5 weeks spec	42.	3.01	{0., 8.}	93.3571	1.96423	newly 5 weeks cumul	210.	147.97	{18., 361.}	66.5286	0.463725	newly 61 weeks cumul	500.	152.31	{18., 361.}	73.37	0.750041
27	newly 3 weeks spec	55.	27.66	{0., 116.}	100.509	1.3243	newly 3 weeks cumul	170.	180.17	{13., 652.}	113.1	0.613005	newly 5 weeks spec	64.	12.1	{0., 39.}	90.875	1.84623	newly 5 weeks cumul	324.	211.7	{13., 833.}	92.858	0.758002	newly 60 weeks cumul	489.	232.19	{13., 887.}	84.2434	0.859104
28	newly 3 weeks spec	52.	36.02	{0., 78.}	78.2692	0.800757	newly 3 weeks cumul	108.	303.41	{86., 712.}	191.676	0.387522	newly 5 weeks spec	42.	11.76	{0., 36.}	82.9048	1.36536	newly 5 weeks cumul	207.	334.82	{105., 771.}	91.8454	0.276353	newly 63 weeks cumul	611.	354.73	{115., 803.}	61.7758	0.444474
29	newly 3 weeks spec	39.	10.73	{0., 24.}	87.2051	1.34731	newly 3 weeks cumul	112.	80.71	{6., 125.}	103.42	0.70188	newly 5 weeks spec	44.	8.01	{0., 17.}	95.3409	2.02534	newly 5 weeks cumul	200.	97.5	{7., 210.}	95.67	0.875972	newly 56 weeks cumul	440.	118.76	{7., 367.}	89.1045	1.13534
30	newly 3 weeks spec	46.	14.89	{0., 23.}	97.6304	1.40951	newly 3 weeks cumul	145.	84.26	{5., 98.}	98.6069	0.785179	newly 5 weeks spec	43.	6.42	{0., 11.}	97.7674	1.96197	newly 5 weeks cumul	245.	100.25	{5., 112.}	96.3796	0.954016	newly 57 weeks cumul	463.	117.53	{5., 197.}	92.8661	1.16832
31	newly 3 weeks spec	17.	13.19	{0., 26.}	115.118	1.23687	newly 3 weeks cumul	48.	126.57	{7., 358.}	221.563	0.436911	newly 5 weeks spec	16.	4.1	{0., 8.}	96.875	1.63993	newly 5 weeks cumul	108.	137.23	{9., 359.}	133.38	0.590188	newly 58 weeks cumul	420.	145.19	{9., 359.}	91.931	1.01503
32	newly 3 weeks spec	35.	27.73	{0., 129.}	123.4	1.28069	newly 3 weeks cumul	65.	190.64	{14., 677.}	243.015	0.44439	newly 5 weeks spec	21.	10.85	{0., 33.}	111.286	1.56871	newly 5 weeks cumul	124.	219.84	{15., 834.}	160.177	0.522271	newly 60 weeks cumul	460.	241.13	{16., 888.}	85.5761	0.808795
33	newly 3 weeks spec	48.	35.51	{0., 75.}	74.1458	0.759088	newly 3 weeks cumul	140.	285.19	{71., 690.}	125.093	0.308341	newly 5 weeks spec	62.	12.82	{0., 31.}	86.8065	1.41691	newly 5 weeks cumul	272.	318.39	{74., 762.}	68.7022	0.289269	newly 63 weeks cumul	628.	343.39	{92., 800.}	65.3933	0.492038
34	newly 3 weeks spec	16.	11.13	{0., 20.}	108.563	1.13187	newly 3 weeks cumul	68.	128.17	{8., 280.}	171.691	0.493026	newly 5 weeks spec	22.	3.18	{0., 6.}	93.3636	1.68146	newly 5 weeks cumul	123.	137.25	{9., 282.}	127.146	0.634378	newly 55 weeks cumul	356.	142.63	{9., 343.}	92.6601	0.957249
35	newly 3 weeks spec	73.	39.2	{0., 75.}	69.863	0.753316	newly 3 weeks cumul	170.	293.03	{89., 752.}	95.4176	0.254509	newly 5 weeks spec	62.	11.57	{0., 31.}	81.3387	1.34559	newly 5 weeks cumul	317.	325.	{106., 790.}	58.6877	0.261001	newly 63 weeks cumul	588.	349.88	{124., 809.}	60.1701	0.410324
36	newly 3 weeks spec	88.	53.03	{2., 113.}	62.7841	0.622689	newly 3 weeks cumul	210.	417.94	{153., 936.}	113.133	0.288997	newly 5 weeks spec	57.	16.67	{0., 40.}	76.8246	1.10996	newly 5 weeks cumul	377.	463.36	{174., 1008.}	59.1247	0.231414	newly 64 weeks cumul	488.	487.13	{179., 1047.}	52.1004	0.25286
37	newly 3 weeks spec	41.	8.33	{0., 10.}	92.9024	1.48854	newly 3 weeks cumul	138.	60.85	{3., 134.}	90.2681	0.928613	newly 5 weeks spec	36.	2.48	{0., 7.}	93.1667	1.97009	newly 5 weeks cumul	224.	67.32	{3., 164.}	87.2232	1.06229	newly 54 weeks cumul	482.	70.81	{3., 165.}	87.0768	1.33235
38	newly 3 weeks spec	82.	30.19	{2., 70.}	80.0366	0.912831	newly 3 weeks cumul	203.	219.56	{36., 524.}	85.468	0.399666	newly 5 weeks spec	63.	9.55	{0., 24.}	89.	1.67605	newly 5 weeks cumul	360.	245.95	{39., 604.}	74.5639	0.518778	newly 62 weeks cumul	525.	268.97	{44., 827.}	73.5905	0.616473
39	newly 3 weeks spec	46.	9.23	{0., 18.}	89.8913	1.60545	newly 3 weeks cumul	116.	94.86	{7., 103.}	106.276	0.646691	newly 5 weeks spec	36.	2.3	{0., 7.}	94.6667	2.01989	newly 5 weeks cumul	202.	101.37	{8., 115.}	96.2723	0.819385	newly 58 weeks cumul	447.	105.86	{10., 123.}	89.2617	1.08465
40	newly 3 weeks spec	52.	52.46	{1., 127.}	75.3077	0.553938	newly 3 weeks cumul	151.	389.18	{127., 888.}	168.013	0.368557	newly 5 weeks spec	41.	17.45	{0., 40.}	72.4634	1.10585	newly 5 weeks cumul	250.	436.74	{148., 1046.}	97.048	0.28487	newly 64 weeks cumul	483.	462.09	{165., 1082.}	54.6894	0.284605
41	newly 3 weeks spec	64.	9.31	{0., 21.}	87.0781	1.44121	newly 3 weeks cumul	223.	146.89	{16., 388.}	72.5964	0.537473	newly 5 weeks spec	76.	1.72	{0., 4.}	97.7368	2.37296	newly 5 weeks cumul	393.	152.44	{16., 396.}	71.5725	0.6966	newly 61 weeks cumul	574.	154.98	{16., 396.}	76.5679	0.833713
42	newly 3 weeks spec	30.	11.65	{0., 25.}	80.5	1.04362	newly 3 weeks cumul	92.	129.46	{17., 276.}	87.2609	0.346526	newly 5 weeks spec	39.	3.48	{0., 9.}	91.641	1.81859	newly 5 weeks cumul	182.	139.23	{17., 338.}	66.489	0.45103	newly 61 weeks cumul	534.	144.17	{17., 344.}	75.7697	0.810097
43	newly 3 weeks spec	35.	6.26	{0., 9.}	90.0571	1.34229	newly 3 weeks cumul	108.	72.45	{16., 69.}	107.528	0.681876	newly 5 weeks spec	36.	2.09	{0., 6.}	94.4167	1.94591	newly 5 weeks cumul	191.	77.94	{19., 117.}	95.3613	0.837421	newly 54 weeks cumul	401.	88.66	{19., 286.}	87.606	1.06307
44	newly 3 weeks spec	64.	31.7	{2., 83.}	80.5625	0.794507	newly 3 weeks cumul	144.	226.32	{34., 599.}	107.278	0.338569	newly 5 weeks spec	60.	10.36	{0., 27.}	88.	1.49289	newly 5 weeks cumul	278.	253.94	{38., 650.}	79.8633	0.436893	newly 62 weeks cumul	520.	279.03	{38., 713.}	73.4212	0.596169
45	newly 3 weeks spec	36.	14.29	{0., 20.}	95.6389	1.11807	newly 3 weeks cumul	140.	109.62	{9., 196.}	105.686	0.666353	newly 5 weeks spec	47.	3.78	{0., 7.}	93.3617	1.92196	newly 5 weeks cumul	246.	120.42	{9., 219.}	94.9268	0.826859	newly 59 weeks cumul	529.	125.2	{9., 242.}	89.2363	1.09456
46	newly 3 weeks spec	25.	7.73	{0., 10.}	98.76	1.54746	newly 3 weeks cumul	61.	83.88	{8., 91.}	121.672	0.432705	newly 5 weeks spec	28.	2.69	{0., 5.}	98.25	2.02521	newly 5 weeks cumul	125.	90.64	{9., 99.}	100.864	0.651933	newly 58 weeks cumul	517.	95.06	{9., 100.}	91.1605	1.17614
47	newly 3 weeks spec	97.	50.17	{4., 103.}	62.1134	0.599398	newly 3 weeks cumul	190.	355.03	{143., 775.}	100.479	0.267122	newly 5 weeks spec	95.	16.87	{0., 37.}	83.2526	1.26648	newly 5 weeks cumul	400.	400.59	{152., 865.}	50.2725	0.238696	newly 64 weeks cumul	577.	426.77	{165., 956.}	49.6274	0.302967
48	newly 3 weeks spec	31.	7.13	{0., 16.}	91.8387	1.46582	newly 3 weeks cumul	100.	79.5	{7., 99.}	98.68	0.627311	newly 5 weeks spec	36.	1.85	{0., 5.}	96.0278	2.12003	newly 5 weeks cumul	184.	84.82	{7., 108.}	92.913	0.831542	newly 58 weeks cumul	364.	87.93	{7., 112.}	89.3489	1.07382
49	newly 3 weeks spec	70.	32.58	{3., 83.}	79.2286	0.829784	newly 3 weeks cumul	184.	239.8	{49., 558.}	92.1957	0.339014	newly 5 weeks spec	55.	10.63	{0., 23.}	88.0545	1.54573	newly 5 weeks cumul	319.	267.74	{51., 636.}	75.8558	0.429374	newly 62 weeks cumul	565.	295.33	{53., 786.}	70.3628	0.562997
50	newly 3 weeks spec	37.	19.33	{0., 41.}	104.405	1.31637	newly 3 weeks cumul	82.	166.16	{26., 556.}	162.902	0.387962	newly 5 weeks spec	52.	7.93	{0., 11.}	93.3269	1.8947	newly 5 weeks cumul	186.	185.68	{27., 686.}	106.473	0.548856	newly 60 weeks cumul	449.	200.98	{31., 862.}	84.0846	0.792963
